# Supplementary material for: Exogenous Melatonin Enhances Dihydrochalcone Accumulation in Lithocarpus litseifolius Leaves via Regulating Hormonal Crosstalk and Transcriptional Profiling
Source: Int J Mol Sci. 2024 Apr 23;25(9):4592. doi: 10.3390/ijms25094592 (PMC11083347; doi:10.3390/ijms25094592)
Supplement: Supplementary file 1 [file ijms-25-04592-s001.zip › Supplementary Figure S1-S3.pdf]

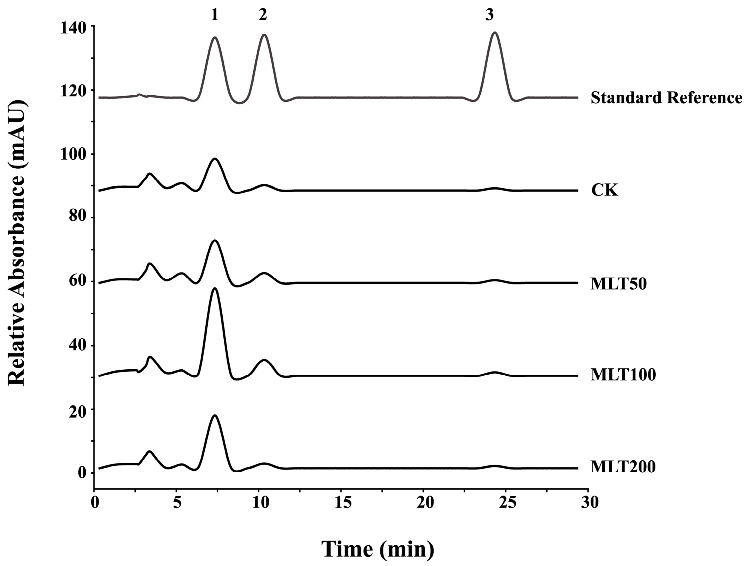

Figure S1. HPLC chromatogram of *L. litseifilus* after MLT treatments

HPLC chromatogram of three representative DHC (**1** phlorizin, **2** trilobatin; **3** phloretin) in *L. litseifilus* after MLT treatments

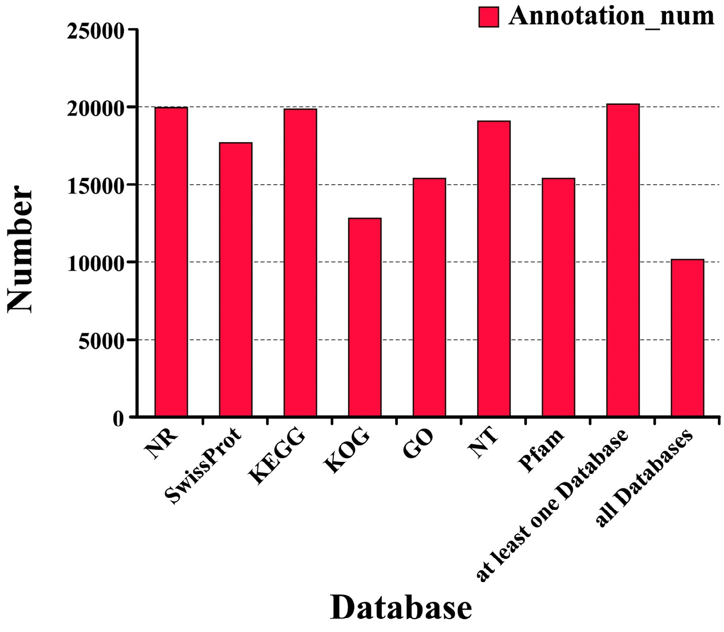

Figure S2 Functional annotations of *L. litseifilus* in databases.

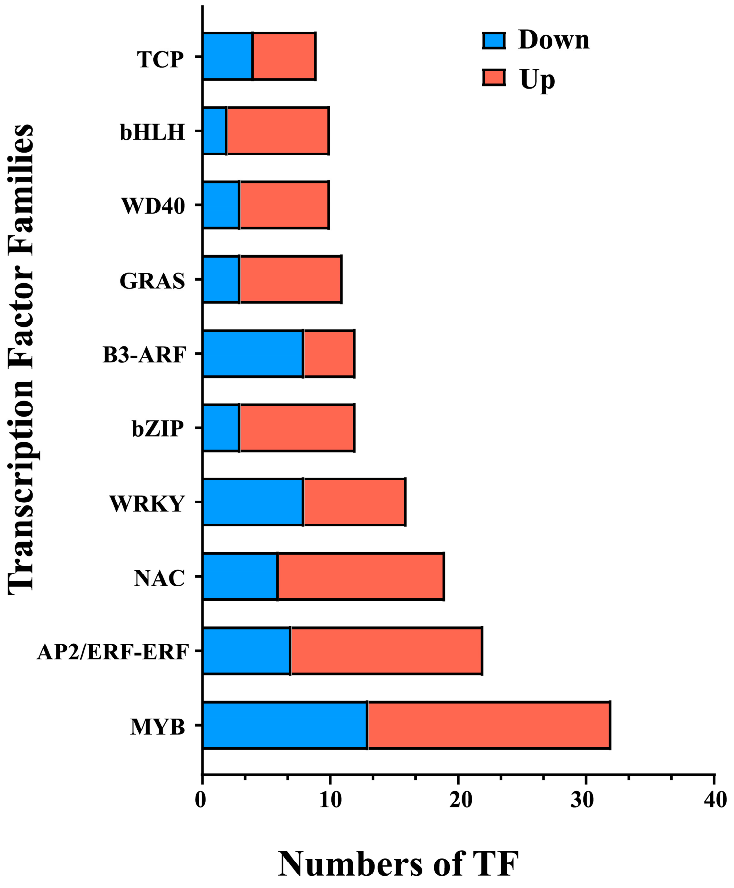

Figure S3 Identification of DEG numbers in TF families.
