# Supplementary material for: Exogenous Melatonin Enhances Dihydrochalcone Accumulation in Lithocarpus litseifolius Leaves via Regulating Hormonal Crosstalk and Transcriptional Profiling
Source: Int J Mol Sci. 2024 Apr 23;25(9):4592. doi: 10.3390/ijms25094592 (PMC11083347; doi:10.3390/ijms25094592)
Supplement: Supplementary file 1 [file ijms-25-04592-s001.zip › Supplementary Table S1-S11.pdf]

Supplementary Table

Table S1 Results statistics of Illumina sequencing data yield

Sample	Error rate (%)	Q20[a] (%)	Q30[b] (%)	GC content[c] (%)	N50 (bp)	Raw reads	Clean reads
CK-1	0.03	96.71	91.14	44.01	2692	67,354,406	66,653,228
CK-2	0.03	97.55	93.16	44.63		59,731,930	56,860,846
CK-3	0.03	96.88	91.55	43.95		64,337,240	63,199,648
MLT100-1	0.03	96.98	91.7	44.19		131,125,870	129,338,674
MLT100-2	0.03	96.74	91.18	43.93		83,678,868	82,604,266
MLT100-3	0.03	96.69	91.15	44.32		68,333,712	66,469,244

a Q20: Percentage of bases that account for more than 20 of Phred values.

b Q30: Percentage of bases that account for more than 30 of Phred values.

c GC content: The sum of base G and C accounts for the percentage of total base number.

Table S2 Information of 20 top enriched KEGG and GO pathways in all comparisons

ID	KEGG Term	Input number	Rich factor	p-value
ko00195	Photosynthesis	23	0.359	0.02548502
ko00944	Flavone and flavonol biosynthesis	2	1	0.05963888
ko04122	Sulfur relay system	4	0.571	0.06526796
ko00920	Sulfur metabolism	11	0.379	0.07378039
ko00785	Lipoic acid metabolism	4	0.5	0.10588440
ko00730	Thiamine metabolism	10	0.357	0.12224470
ko00230	Purine metabolism	27	0.297	0.14681770
ko03060	Protein export	15	0.319	0.15164980
ko00966	Glucosinolate biosynthesis	4	0.444	0.15502220
ko00460	Cyanoamino acid metabolism	20	0.303	0.16454370
ko00600	Sphingolipid metabolism	14	0.318	0.16539250
ko00999	Biosynthesis of various plant secondary metabolites	18	0.305	0.17243980
ko04141	Protein processing in endoplasmic reticulum	66	0.269	0.19359140
ko00310	Lysine degradation	15	0.306	0.19666060
ko00260	Glycine, serine and threonine metabolism	29	0.284	0.20041340
ko01040	Biosynthesis of unsaturated fatty acids	10	0.323	0.20594550
ko00941	Flavonoid biosynthesis	9	0.321	0.22684890
ko00440	Phosphonate and phosphinate metabolism	3	0.429	0.23171100
ko00190	Oxidative phosphorylation	34	0.274	0.24565130
ko00904	Diterpenoid biosynthesis	2	0.5	0.25207070

GO_accession	Description	Term_type	p-value	DEG_item	DEG_list	Bg_item
GO:0005199	structural constituent of cell wall	Molecular Function	5.32E-05	25	2775	48
GO:0031072	heat shock protein binding	Molecular Function	0.00038978	7	2775	8
GO:0043531	ADP binding	Molecular Function	0.00074925	93	2775	276
GO:0005509	calcium ion binding	Molecular Function	0.00117946	44	2775	115
GO:0005516	calmodulin binding	Molecular Function	0.00134142	16	2775	31
GO:0009341	beta-galactosidase complex	Cellular Component	0.00059352	5	2775	5
GO:0016759	cellulose synthase activity	Molecular Function	0.00225208	17	2775	35
GO:0016760	cellulose synthase (UDP-forming) activity	Molecular Function	0.00225208	17	2775	35
GO:0022836	gated channel activity	Molecular Function	0.00343044	18	2775	39
GO:0004601	peroxidase activity	Molecular Function	0.00404349	11	2775	20
GO:0016772	transferase activity, transferring phosphorus-containing groups	Molecular Function	0.00635544	265	2775	927
GO:0032403	protein complex binding	Molecular Function	0.00639731	29	2775	75
GO:0016684	oxidoreductase activity, acting on peroxide as acceptor	Molecular Function	0.00658467	11	2775	21
GO:0015276	ligand-gated ion channel activity	Molecular Function	0.00798472	13	2775	27
GO:0022834	ligand-gated channel activity	Molecular Function	0.00798472	13	2775	27
GO:0035251	UDP-glucosyltransferase activity	Molecular Function	0.01119282	20	2775	49
GO:0008017	microtubule binding	Molecular Function	0.01153439	13	2775	28
GO:0005216	ion channel activity	Molecular Function	0.01171828	25	2775	65
GO:0015267	channel activity	Molecular Function	0.01171828	25	2775	65
GO:0022803	passive transmembrane transporter activity	Molecular Function	0.01171828	25	2775	65

Table S3 Distribution of the number of DEGs in the GO database

Category	Pathway	MLT 100 vs CK
Biological Process	metabolic process	1051
	cellular process	1011
	single-organism process	770
	organic substance metabolic process	770
Cellular Component	membrane	354
	cell	286
	cell part	286
Molecular Function	binding	1713
	catalytic activity	1165
	heterocyclic compound binding	757

Table S4 DEGs associated with the antioxidant enzymes

	Genes	MLT100 vs CK (FPKM mean)
Antioxidant enzymes	*SOD*(42812/f3p0/887)	1.51
	CAT(29712/f5p0/1810)	6.33
	POD(18736/f4p0/2391)	6.22
	POD(22158/f2p0/2190)	2.11
	POD(42497/f2p0/930)	1.92
	POD(35061/f5p0/1508)	1.78
	POD(39139/f2p0/1309)	1.68
	POD(19285/f5p0/2355)	1.57

Table S5 Effect of melatonin on phytohormone contents (ng/g FW) in *L. litseifilus* leaves

		CK-1	CK-2	CK-3	MLT-100-1	MLT-100-2	MLT-100-3	CK mean	MLT100 mean
SA	SA	248.30	251.46	238.67	319.32	324.92	347.05	246.14±6.66	330.43±14.66
	SAG	7704.99	9237.73	8539.86	7030.32	6953.74	6847.14	8494.19±767.39	6943.73±92
JA	H2JA	0.00	0.00	0.00	1.14	0.79	0.98	0±0	0.97±0.18
	JA-Phe	0.00	0.00	0.00	0.48	0.29	0.60	0±0	0.46±0.16
	MeJA	15.32	3.26	1.94	9.88	11.35	14.47	6.84±7.37	11.9±2.35
	OPDA	47.39	62.86	74.75	73.02	73.82	87.90	61.67±13.72	78.25±8.37
	JA-ILE	13.82	15.52	14.90	9.30	9.73	8.64	14.75±0.86	9.22±0.55
	JA-Val	0.96	0.96	0.79	0.52	0.55	0.48	0.9±0.1	0.52±0.04
	JA	46.38	49.64	46.36	27.97	26.96	24.61	47.46±1.89	26.51±1.72
ETH	ACC	14.77	17.01	17.96	21.56	22.39	19.99	16.58±1.64	21.31±1.22

Values are means±SDs (rightmost four columns).

Table S6 DEGs associated with the biosynthesis of three phytohormone types

	Genes	MLT100 vs CK (FPKM mean)
SA	*OPR3*(43195/f2p0/743)	**3.44**
	AOC4(28958/f2p0/1847)	**1.63**
	SGT1(36373/f3p0/1476)	**1.54**
JA	*JAZ*(38589/f3p0/1249)	**12.61**
	JAZ(36112/f3p0/1495)	**2.12**
	JAZ(38011/f2p0/1379)	**1.79**
ETH	*ERF105*(34493/f9p0/1534)	**3.89**
	ERF105(33916/f3p0/1618)	**3.38**
	ERF102(36892/f4p0/1386)	**2.81**
	ERF17(41133/f22p0/1097)	**2.40**
	ABR1(32460/f8p0/1668)	**2.33**
	ERF105(26384/f2p0/1971)	**2.15**
	ERF4(38181/f3p0/1348)	**1.94**
	ERF4(24979/f4p0/2034)	**1.91**
	ERF11(42043/f2p0/1024)	**1.86**
	ERF78(41901/f7p0/1019)	**1.83**
	EIN2(940/f2p0/5032)	**1.62**
	RAP21(42090/f7p0/1001)	**1.61**
	RAP24(20073/f5p0/2243)	**1.55**
	ETR1(13983/f3p0/2719)	**1.53**
	WRI1(32246/f5p0/1664)	**1.51**

The numbers represent the fold change values. Numbers in red represent up-expressed by at least 1.5-folds, while numbers in green represent down-expressed by at least 1.5-folds.

Table S7 DEGs associated with the MYB, bHLH, WD40, and WRKY TFs

	Genes	MLT100 vs CK (FPKM mean)
MYB	*MYB3R-1* (6026/f2p0/3497)	3.50
	MYB4 (42638/f2p0/900)	2.61
	MYB-TT2 (39030/f3p0/1305)	1.56
	MYB36 (30131/f2p0/1791)	1.51
	POPTR (20601/f2p0/2280)	6.87
	POPTR (18275/f3p0/2364)	6.02
	RVE1 (18537/f5p0/2403)	3.26
	LHY (9957/f5p0/2980)	3.22
	LHY (7701/f2p0/3312)	3.15
	RVE7 (23013/f2p0/2162)	2.89
	RVE8 (39010/f2p0/1310)	2.52
	LHY (10618/f6p0/2985)	2.09
	NCOR1 (289/f3p0/5996)	2.07
	LHY (9897/f7p0/3021)	1.90
	SRM1 (32925/f2p0/1672)	1.79
	RVE7 (23202/f3p0/2141)	1.75
	RID2 (14293/f2p0/2695)	1.72
	MYB1R1 (36582/f5p0/1454)	1.69
	RVE1 (19340/f2p0/2372)	1.61
bHLH	*ICE1* (25728/f3p0/1957)	4.90
	bHLH130 (17136/f2p0/2528)	2.49
	bHLH79 (35577/f3p0/1520)	2.42
	bHLH93 (38081/f3p0/1365)	2.30
	bHLH35 (38525/f2p0/1344)	2.17
	PIF3 (14202/f2p0/2702)	2.14
	bHLH128 (29393/f3p0/1828)	2.13
	bHLH128 (15009/f2p0/2651)	1.83
WD40	*WDR76* (31381/f2p0/1736)	3.30
	WDR20 (20462/f2p0/2303)	2.92
	WDAN11 (33576/f3p0/1634)	2.29
	WDAN11 (30394/f2p0/1786)	2.29
	WDR20 (4813/f3p0/3657)	2.02
	WDR43 (18724/f10p0/2389)	1.80
	WDR26 (22794/f3p0/2153)	1.79
WRKY	*WRK24*(20376/f13p0/2273)	11.10
	WRK70(33728/f2p0/1597)	9.79
	WRKY7(36183/f2p0/1488)	3.74
	WRKY2(13386/f7p0/2732)	1.88
	WRK72(25547/f2p0/2006)	1.88
	WRK20(21711/f4p0/2211)	1.78
	WRKY3(21493/f28p0/2216)	1.53
	WRK31(18990/f4p0/2375)	1.52

Table S8 DEGs associated with the flavonoid biosynthesis

Pathway	Genes	MLT100 vs CK (FPKM mean)
flavonoid biosynthesis	*PAL*(15514/f17p0/2519)	1.50
	4CL(34202/f4p0/1578)	5.33
	4CL(15232/f2p0/2628)	2.16
	4CL(34871/f3p0/1566)	2.05
	4CL(26318/f3p0/1973)	1.65
	C4H(27236/f3p0/1923)	5.19
	C4H(27875/f12p0/1831)	1.62
	HCT(32367/f20p0/1673)	1.70
flavone and flavonol biosynthes	*CHI*(42775/f2p0/868)	2.62
	FlS(37677/f7p0/1394)	3.47
phenylpropanoid biosynthesis	*CADH9*(37130/f7p0/1408)	8.14
	BGL45(30760/f2p0/1685)	4.22
	HIUH(24757/f3p0/2046)	2.13
	BGL11(29948/f26p0/1732)	2.10
	PER64(39139/f2p0/1309)	1.68
	BGL12(14915/f2p0/2647)	1.61
	BGL11(23810/f2p0/2123)	1.60
	CADH1(34122/f2p0/1615)	1.59

Table S9 DEGs associated with the UGTs

	Genes	MLT100 vs CK (FPKM mean)
UGT	*UGT91A1*(30673/f2p0/1775)	5.83
	UGT709C2(29880/f3p0/1805)	4.25
	UGT73C6(28609/f2p0/1803)	3.81
	UGGT(1122/f2p0/4897)	3.75
	UGT73C6(29340/f2p0/1828)	3.65
	UGT76F1(32451/f4p0/1653)	2.52
	UGT73B4(28849/f3p0/1848)	2.43
	UGT71K1(19493/f4p0/2347)	2.42
	UGT71K1(39345/f2p0/1284)	2.40
	UGT75L6(34509/f4p0/1580)	2.39
	UGT87A1(33556/f3p0/1639)	2.35
	UGT85A24(33704/f2p0/1626)	2.16
	UGT83A1(34105/f8p0/1569)	2.07
	UGT80A2(22882/f2p0/2148)	1.94
	UGT74E1(33569/f3p0/1598)	1.93
	UGT709C2(34005/f3p0/1610)	1.85
	UGT92A1(25736/f4p0/2011)	1.62
	UGT83A1(25357/f2p0/2046)	1.57
	UGT709C2(32233/f2p0/1732)	1.56
	SGT1B(36373/f3p0/1476)	1.54
	UGT88A1(32492/f35p0/1640)	1.53

Table S10 List of all kits used in this study

Kit name	Kit model	Kit website	Manufacturer
Hydrogen Peroxide(H_2O_2) Content Assay Kit	BC3590	https://www.solarbio.com/goodsInfo?id=38920	Solarbio(China)
Malondialdehyde(MDA) Content Assay Kit	BC0020	https://www.solarbio.com/goodsInfo?id=6098	Solarbio(China)
Superoxide Dismutase(SOD) Activity Assay Kit	BC0175	https://www.solarbio.com/goodsInfo?id=9124	Solarbio(China)
Catalase(CAT) Activity Assay Kit	BC0200	https://www.solarbio.com/goodsInfo?id=6114	Solarbio(China)
Peroxidase(POD) Activity Assay Kit	BC0090	https://www.solarbio.com/goodsInfo?id=6104	Solarbio(China)
Glutathione Reductases(GR) Activity Assay Kit	BC1160	https://www.solarbio.com/goodsInfo?id=6187	Solarbio(China)
Phenylalnine Ammonialyase(PAL) Activity Assay Kit	BC0215	https://www.solarbio.com/goodsInfo?id=9128	Solarbio(China)
Cinnamic acid 4-hydroxylase(C4H) Activity Assay Kit	BC4085	https://www.solarbio.com/goodsInfo?id=51045	Solarbio(China)
4-Coumarate CoA Ligase(4CL) Activity Assay Kit	BC4225	https://www.solarbio.com/goodsInfo?id=64616	Solarbio(China)
RNAprep Pure Plant Plus Kit (Polysaccharides&Polyphenolics-rich)	DP441	https://www.tiangen.com/content/details_40_21390.html	Tiangen(China)
TransScript® All-in-One First-Strand cDNA Synthesis SuperMix for qPCR (One-Step gDNA Removal)	AT341-01	https://www.transgenbiotech.com/rt_pcr/transscript_all_in_one_first_strand_cdna_synthesis_for_qpcr.html	TransGen Biotech(China)
SuperReal PreMix Color (SYBR Green	FP215-02	https://www.tiangen.com/content/details_40_21225.html	Tiangen(China)

Table S11 Sequence of primer pairs used in qRT-PCR analysis.

**Name**	**Gene ID**	**Orientation**	**Sequence(5'-3')**
PAL	15514/f17p0/2519	Forward	GCATAGGCGAACCAAGCAA
		Reverse	CAGCAATGTAGGATAGAGGGACA
4CL	34202/f4p0/1578	Forward	ACTGCGAACCCGTTTTACACT
		Reverse	AACCCTCCACTGGCGACTC
UGT71K1	19493/f4p0/2347	Forward	TGATGTCTTGCCAGAAGGTTTC
		Reverse	CCTCCTATTGCTTTGTGAGCC
SOD	42812/f3p0/887	Forward	GTGAAGGCTGTGGCTGTTCTT
		Reverse	TTTGTTAGCAGGATTAAAATGTGGT
POD	22158/f2p0/2190	Forward	GCTATGACCCCGCTTACGA
		Reverse	CGGTGAAGAGGAACTTGGATG
MYB-TT2	39030/f3p0/1305	Forward	TGAAATCAAGAACTATTGGAACACC
		Reverse	TGATGACATTGACAGCCTTCG
bHLH130	17136/f2p0/2528	Forward	TATGATCTCGGATAATATCACTGGTC
		Reverse	TCAGCAATGCTTCGTGGGT
SGT1	36373/f3p0/1476	Forward	CCAGCAGATTAGCACGATTACAT
		Reverse	AGCTTTCGCAAGACGGATTT
JAZ	38589/f3p0/1249	Forward	TAAGGAGGAGGTCCCAAAGATT
		Reverse	CACGATTACTTGTCCACCATAGAA
GAPDH	reference genes	Forward	CCTTCCGTGTTCCTACCCCCAA
		Reverse	TAGCCCAAGATGCCCTTCAGT
